# Targeting mosquito FREP1 with a fungal metabolite blocks malaria transmission

**DOI:** 10.1038/srep14694

**Published:** 2015-10-06

**Authors:** Guodong Niu, Bin Wang, Genwei Zhang, Jarrod B. King, Robert H. Cichewicz, Jun Li

**Affiliations:** 1 Department of Chemistry and Biochemistry, University of Oklahoma, 101 Stephenson Parkway, Norman, OK 73019, USA

## Abstract

Inhibiting *Plasmodium* development in mosquitoes will block malaria transmission. Fibrinogen-related protein 1 (FREP1) is critical for parasite infection in *Anopheles gambiae* and facilitates *Plasmodium* invasion in mosquitoes through interacting with gametocytes and ookinetes. To test the hypothesis that small molecules that disrupt this interaction will prevent parasites from infecting mosquitoes, we developed an ELISA-based method to screen a fungal extract library. We obtained a candidate fungal extract of *Aspergillus niger* that inhibited the interaction between FREP1 and *P*. *falciparum* infected cells by about 92%. The inhibition specificity was confirmed by immunofluorescence assays. Notably, feeding mosquitoes with the candidate fungal extract significantly inhibited *P. falciparum* infection in the midgut without cytotoxicity or inhibition of the development of *P. falciparum* gametocytes or ookinetes. A bioactive natural product that prevents FREP1 from binding to gametocytes or ookinetes was isolated and identified as *P*-orlandin. Importantly, the nontoxic orlandin significantly reduced *P. falciparum* infection intensity in mosquitoes. Therefore, disruption of the interaction between FREP1 and parasites effectively reduces *Plasmodium* infection in mosquitoes. Targeting FREP1 with small molecules is thus an effective novel approach to block malaria transmission.

*Plasmodium* parasites, transmitted by *Anopheline* mosquitoes, cause more than 198 million clinical cases and over 584,000 deaths per year according to the World Malaria Report 2014 from the World Health Organization^1^. Approximately 90% of malaria-caused deaths occur in Africa. The human malaria pathogen, *P. falciparum*, has a complex life cycle involving humans and mosquitoes. Within red blood cells in human blood, some asexual stage parasites differentiate into sexual stage gametocytes that can infect mosquitoes. Since the passage of the *Plasmodia* through the vector mosquitoes is an obligatory step for malaria transmission, using pesticides to control the mosquito population has traditionally been an effective method to control malaria. However, the limited number of molecular targets inhibited by insecticides^2^,^3^ and the limited types of insecticides^2^,^4^,^5^,^6^ have accelerated the spread of insecticide-resistance^7^ in mosquito populations^8^,^9^. More strikingly, there have been very few novel insecticides brought to the market in the past 30 years^10^. To combat malaria, the public health community desperately needs new strategies.

Blocking *Plasmodium* infection in mosquitoes will stop malaria transmission. To date, research efforts have focused on drugs that kill parasites in the blood stage^11^,^12^,^13^, while no compounds have been developed that target mosquito proteins and block malaria transmission without killing the mosquitoes. After mosquitoes take an infected blood meal, gametocytes differentiate into mobile ookinetes that invade mosquito midguts to form oocysts. About ten days later, these oocysts become mature and release sporozoites into hemolymph. The sporozoites in salivary glands are injected into the next host to start another cycle of infection. This *Plasmodium* invasion of mosquitoes depends on the interactions between parasites and mosquito molecules. Many mosquito proteins involved in *Plasmodium* infection have been identified^14^,^15^,^16^ including Tep1 in hemolymph, a complement-like protein of the mosquito’s innate immunity that inhibits malaria infection^17^. APL1C is another protein in the hemolymph, which forms a complex with LRIM1 and inhibits the *Plasmodium* infection in mosquito^18^,^19^. *Plasmodium* parasites are also known to use midgut proteins to facilitate their invasion. Antibodies against mosquito midgut extracts have been shown to inhibit the development of the rodent malaria parasite, *P. berghei* and the human parasites, *P. falciparum* and *P. vivax* in several *Anopheles* mosquitoes^20^. In particular, antibodies against anopheline alanyl aminopeptidase N (AnAPN1)^21^ and carboxypeptidases B^22^ have been reported to block *Plasmodium* infection^23^. Notably, targeting these mosquito proteins with small molecules to block malaria transmission has not yet been reported. If such candidate compounds were to be identified, they could be administered in combination with anti-malaria drugs to block malaria transmission or alternatively the compounds could be sprayed outdoors or on bednets to block malaria transmission.

We recently identified fibrinogen-related protein 1 (FREP1) from *Anopheles gambiae* that is critical for *Plasmodium* invasion in mosquitoes. A mutation in FREP1 is associated with clinically circulating *P. falciparum* infection intensity (the number of oocysts per mosquito midgut) in wild mosquitoes and silencing FREP1 renders most if not all *An. gambiae* free from *P. berghei*^16^ infection and significantly decreases *P. falciparum* infection in *An. gambiae* while antibodies against FREP1 inhibit *P. falciparum* infection in *An. gambiae* mosquitoes^24^. Therefore, FREP1 protein is an excellent target to block malaria transmission. FREP1 is a member of the fibrinogen-related protein family (FREP, also known as fibrinogen domain immunolectins (FBNs)) that contains a conserved fibrinogen-like domain consisting of approximately 200 amino acids 25,26. In mammals, fibrinogens are involved in blood coagulation. In invertebrates, FREPs function as pattern recognition receptors, and are capable of binding to bacteria, fungi or parasites^27^. FREP1 is in the mosquito peritrophic matrix and facilitates *Plasmodium* infection through binding to gametocytes or ookinetes^24^. We hypothesize that disruption of the interaction between FREP1 and parasites with small molecules may inhibit parasite infection in mosquitoes.

To test this hypothesis, we developed an ELISA-based approach to screen a small molecule library to find candidate drugs that can disrupt FREP1-parasite interaction. Instead of screening a library of synthetic compounds, we started by screening a fungal extract library, because each extract contains dozens to hundreds of small molecules^28^ that can better demonstrate the proof of concept. We successfully identified a candidate extract from fungal isolate Chapel SA-3 and verified its effect on *P. falciparum* infection in *An. gambiae* mosquitoes. Moreover, purified compound *P*-orlandin was obtained from an extract derived from isolate Chapel SA-3 and it was shown to effectively inhibit *P. falciparum* infection in mosquitoes.

## Results

### Insect cell-expressed FREP1 protein and its interaction with *P. falciparum*

**Figure 1:**
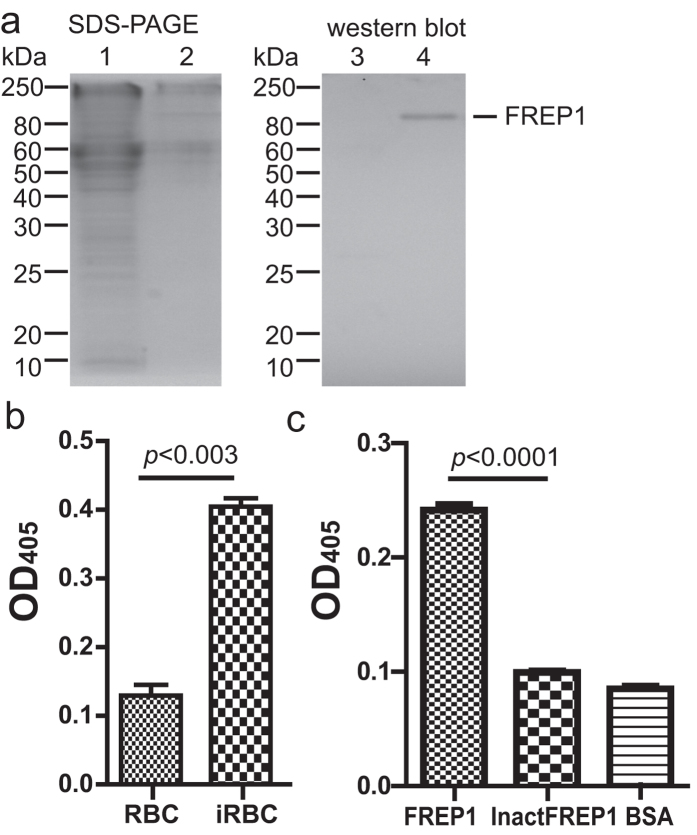
Insect cell-expressed FREP1 protein and its interaction with iRBC lysate detected by ELISA. (**a**) The FREP1 was expressed and secreted from High Five cells, determined by 12% SDS-PAGE (left) and western blot assay (right). This result also demonstrated that anti-FREP1 antibody could specifically recognize FREP1. Lanes: 1,3: cell lysate; 2,4: culture medium. (**b**) ELISA signals were significantly different between *Plasmodium falciparum*-infected red blood cell (iRBC) lysate and uninfected RBC lysate. The lysate of iRBC and uninfected human RBC were used to coat the ELISA plate, followed by sequential incubation with recombinant insect cell expressed FREP1 protein, 1^st^ antibody, alkaline phosphatase-conjugated 2^nd^ antibody. The samples were developed by the addition of 100 μL of pNPP and OD_405_ reading. The retained FREP1 in iRBC lysate-treated wells was significantly higher than in uninfected RBC lysate (*p* < 0.0002). (**c**) When the heat inactivated FREP1 protein (InactFREP1) replaced the functional FREP1, the binding between FREP1 and iRBC lysate disappeared (*p* < 0.001).

Because FREP1 expressed in *E. coli* forms inclusion bodies and lacks post-translational modification, we used an insect cell expression system to generate functional FREP1 that is similar to endogenous FREP1. The endotoxin-free pIB-FREP1 plasmid was used to express FREP1 in insect cells using protein-free medium. We found that FREP1 was specifically detected in the culture medium of the insect High Five cells transfected with pIB-FREP1 DNA but not in the cell lysate (Fig. 1a), confirming that FREP1 is a secreted protein. The western blotting result also demonstrates that the anti-FREP1 polyclonal antibody^24^ can specifically recognize FREP1.

Next we developed an ELISA approach to detect the interaction between the insect cell-expressed FREP1 protein and *P. falciparum* parasites. The *P. falciparum* were cultured for 15–17 days and the culture contained 20%–30% asexual stage parasites (e.g. rings, trophozoites, schizonts), 5%–10% gametocytes, and many merazoites (free parasites). ELISA plates were coated with this late cultured *P. falciparum*-infected red blood cell (iRBC) lysate, followed by incubation with FREP1, and the retained FREP1 was specifically recognized by anti-FREP1 antibodies and quantified using ELISA at OD_405_. The uninfected human red blood cell (RBC) lysate was used as a negative control. The results (Fig. 1b) showed that the OD_405_ of iRBC lysate coated wells were significantly (p < 0.003) higher than that of uninfected RBC lysate coated wells, indicating that the ELISA approach can effectively distinguish positives from negatives. To demonstrate that the binding signal was caused by functional FREP1, we inactivated FREP1 (65 °C for 15 min), and used it or BSA to substitute FREP1. The heat-inactivated FREP1 generated a significantly lower ELISA signal (Fig. 1c) compared to the functional FREP1 (p < 0.0001) however similar to the negative control (BSA), demonstrating the interaction between FREP1 and iRBC is mediated by the functional FREP1.

### Screening fungal extracts that disrupt FREP1-iRBC lysate interaction by an ELISA-based approach

**Figure 2:**
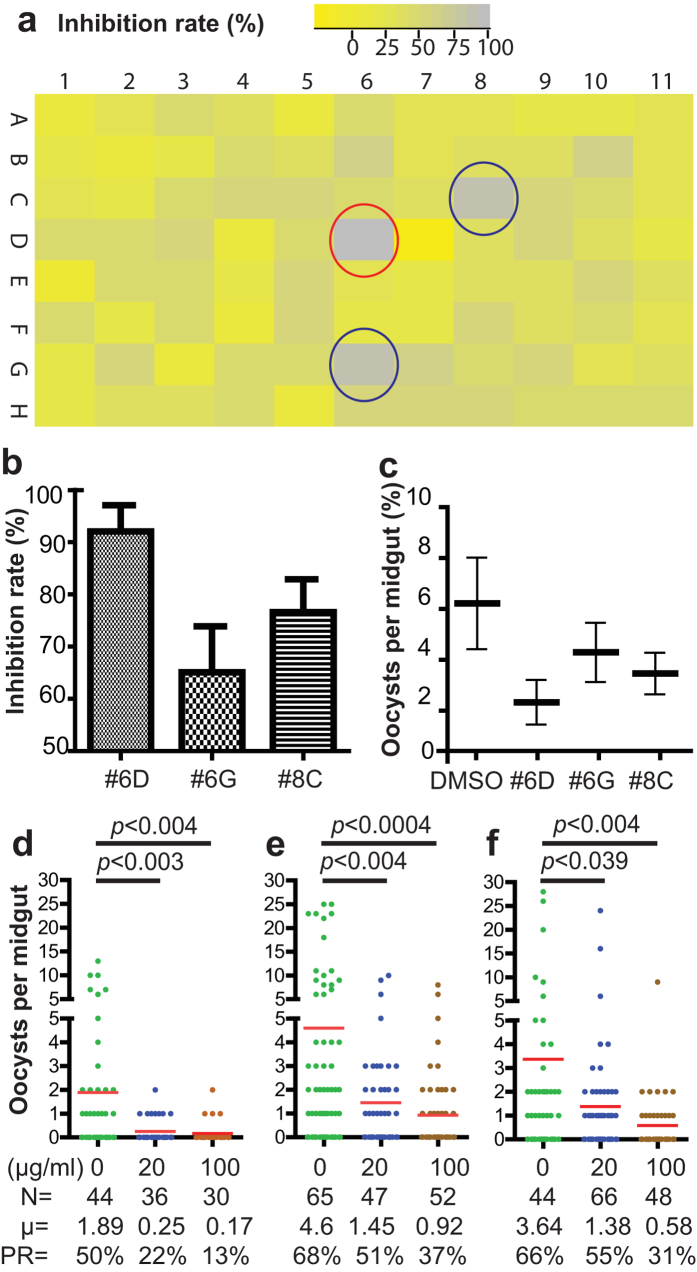
Identification of fungal extracts that disrupt FREP1-iRBC lysate interaction and interfere with *P. falciparum* infection in mosquitoes. (**a**) Screening fungal extracts that inhibit FREP1 binding to iRBC lysate by ELISA. Each rectangle represents an individual fungal extract and the color represents the inhibition rate. Three fungal extracts that had high inhibition rates are marked with circles. The assays were repeated twice and the graph was generated by R-Project software. The fungal extract (marked with red circle) had the highest inhibition rate. (**b**) Validation of the identified fungal extracts by ELISA using four replicates. (**c**) The number of oocyst in mosquito midguts co-ingesting *P. falciparum* gametocytes with 20 μg/mL candidate fungal extracts. (**d–f**) The candidate fungal extract (#6D from isolate Chapel SA-3) significantly inhibited *P. falciparum* infection in mosquito midguts and the inhibition displayed a dose-dependent pattern. The assays were repeated independently three times. The results showed significantly fewer oocysts developed in *An. gambiae* midguts treated with Chapel SA-3 extract than treated with DMSO (p < 0.05). N: the number of mosquitoes for each treatment; μ: the mean oocysts per midgut; PR: infection prevalence in mosquitoes.

Since FREP1 promotes *Plasmodium* invasion through binding to *P. falciparum* gametocytes or ookinetes^24^, we hypothesize that small molecules interfering with FREP1-parasite interaction might be good candidates to inhibit parasite infection in mosquitoes. Using the ELISA assay, we screened a library of crude extracts from various fungi. A fungal extract was judged to have completely inhibited the binding between FREP1 and iRBC if the ELISA signals were decreased to the level of the uninfected RBC control (gray color in Fig. 2a). After screening a plate of fungal extracts (N = 88), we found three fungal extracts (marked with circles in Fig. 2a) that exhibited >75% inhibition rates. The inhibitory activities of the three candidate extracts against FREP1-iRBC lysate interaction were reconfirmed in biologically replicates (N = 4) before further testing occurred. Dimethyl sulfoxide (DMSO) was used as a negative control. The results (Fig. 2b) show that candidate extracts #6D, #6G, and #8C inhibited 92%, 64%, and 75% of FREP1-iRBC lysate interaction, respectively, compared to the negative control. Next, we determined their effects on *P. falciparum* infection in mosquitoes. For each test, a 5 μL sample of fungal extract (2 mg/mL) in DMSO was added into 495 μL human blood containing 0.2% sexual stage V gametocytes. The results from standard membrane feeding assays (SMFA) indicated that all three candidates reduced the number of oocysts in mosquitoes (Fig. 2c) compared to the DMSO control. Notably, the *in vivo* infection inhibition rates of three candidates (Fig. 2c) matched their *in vitro* inhibition rates on FREP1-iRBC lysate interaction (Fig. 2b). Because fungal extract #6D had ~92% inhibition rate (marked with red circle in Fig. 2a) and was most effective in reducing *P. falciparum* infection in mosquito midguts, we focused on this sample to demonstrate the principle of our proposed approach. Extract #6D was obtained from an isolate designated “Chapel SA-3” and it is noted as such throughout this manuscript.

### Chapel SA-3 significantly interfered with *P. falciparum* infection in *An. gambiae* mosquitoes in a dose-dependent pattern

Next we determined whether the candidate fungal extract inhibited *P. falciparum* infection in *An. gambiae* mosquitoes in a dose-dependent pattern. Human blood containing 0.2% stage V gametocytes supplemented with 1% of DMSO containing 0, 20 or 100 μg/mL of the Chapel SA-3 was used respectively to feed female mosquitoes by SMFA. After seven-day post infection, the number of oocysts in bloodfed mosquito midguts was examined. Significantly fewer oocysts were developed in the mosquito midguts treated with Chapel SA-3 than the control group (p < 0.05, Fig. 2d–f). As shown in Fig. 2d, the mean numbers of oocysts in mosquitoes treated with 0, 20 and 100 μg/mL fungal extract groups were 1.89, 0.25 and 0.17, and the prevalence rates of the three treatments were 50%, 22% and 13%, respectively. Three independent replicates showed consistent results and indicated that Chapel SA-3 significantly reduced the *P. falciparum* infection in *An. gambiae*. Evidently, interference of *P. falciparum* infection by Chapel SA-3 showed a dose-dependent pattern, e.g. increasing the Chapel SA-3 extract concentration resulted in decreased *P. falciparum* infection in mosquitoes (Fig. 2d–f).

### The specificity of Chapel SA-3 on FREP1-parasite interaction

**Figure 3:**
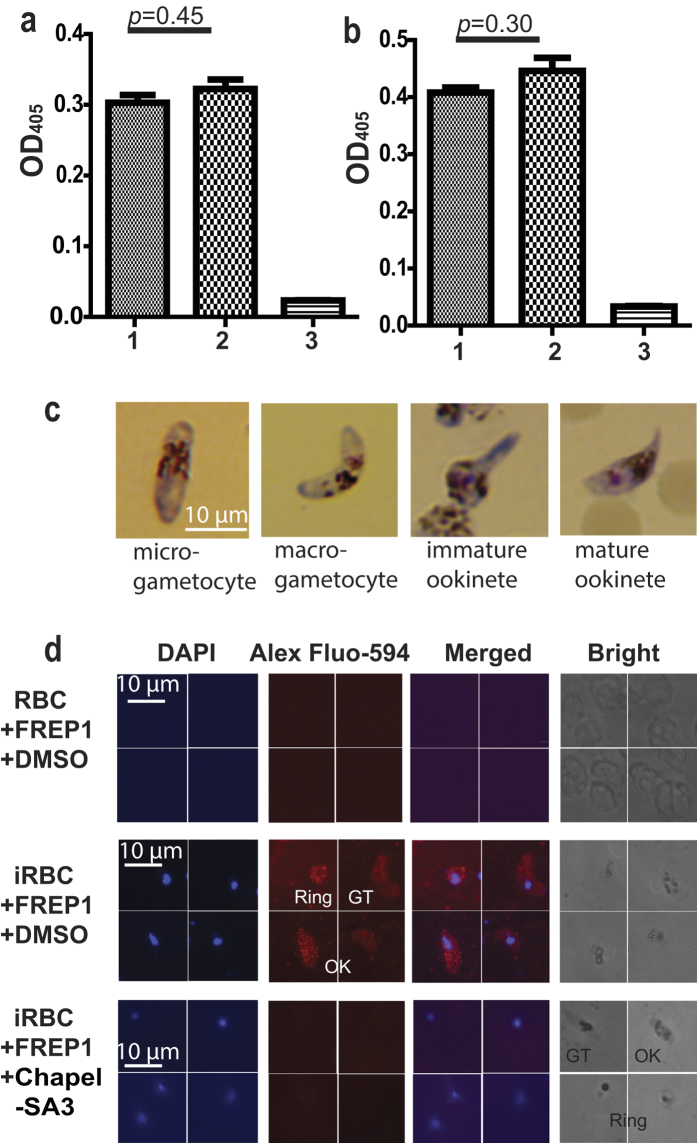
Chapel SA-3 extract specifically prevents FREP1 from binding *P. falciparum* rings, gametocytes and ookinetes. (**a**) The extract from isolate Chapel SA-3 did not affect the interaction between FREP1 and anti-FREP1 polyclonal antibody nor 1^st^ antibody and 2^nd^ antibody determined by ELISA. Treatments: 1: FREP1 (7.5 μg/mL) plus DMSO (1%); 2: FREP1 (7.5 μg/mL) plus Chapel SA-3 extract (100 μg/mL); 3: BSA (7.5 μg/mL) plus DMSO (1%). (**b**) The extract from isolate Chapel SA-3 did not affect the unrelated molecule-molecule interaction. Treatments: 1: unrelated His-tagged protein (7.5 μg/mL) plus DMSO (1%); 2: unrelated His-tagged protein (7.5 μg/mL) plus Chapel SA-3 (100 μg/mL); 3: BSA (7.5 μg/mL) plus DMSO (1%). (**c**) The morphology of gametocytes and ookinetes stained by the Giemsa staining. (**d**) The extract from isolate Chapel SA-3 inhibited the binding between FREP1 protein and *P. falciparum* parasites shown by IFA. The first and second column detected cell nuclei stained with DAPI and FREP1, respectively. Merging column one and two generated the third column that shows the co-localization of *P. falciparum* (nuclei) and FREP1 binding. The 4^th^ column shows the bright views of the cells. The uninfected RBC did not bind FREP1 (1^st^ row)’; *P. falciparum* iRBC rings, gametocytes (GT), and ookinetes (OK) interacted with FREP1 (2^nd^ row); and such interactions were disrupted by Chapel SA-3 (3^rd^ row).

To analyze whether Chapel SA-3 prevented anti-FREP1 antibody from binding FREP1 or prevented secondary antibody binding, we coated ELISA plates with the FREP1 and incubated anti-FREP1 with Chapel SA-3, 2^nd^ antibody, and other ELISA reagents sequentially. The DMSO was used as a negative control. Results showed no significant difference (*p* = 0.45) between the negative control (Fig. 3a, column 1), and the Chapel SA-3 treated sample (Fig. 3a, column 2). The OD_405_ values of both negative control and Chapel SA-3 treated sample were much higher than background (Fig. 3a, column 3). These results support the fact that Chapel SA-3 does not inhibit the interaction between FREP1 and anti-FREP1 antibody or anti-FREP1 antibody and the secondary antibody. In addition, the effect of Chapel SA-3 on unrelated molecule-molecule interaction was also tested using an anti-His monoclonal antibody binding unrelated His-tagged protein. Coating an unrelated His-tagged protein onto a plate and detecting with anti-His monoclonal antibody showed similar results (*p* = 0.30) between Chapel SA-3 treated samples (Fig. 3b, column 2) and the control (DMSO treated, Fig. 3b, column 1). The results also showed that the OD_405_ values of both the DMSO-treated and Chapel SA-3 treated sample were much higher than the background (Fig. 3b, column 3). These data collectively show that Chapel SA-3 specifically disrupts the interaction between FREP1 and parasites.

Furthermore, we analyzed the specificity of Chapel SA-3 on the FREP1-*Plasmodium* interaction using indirect immunofluorescence assay (IFA). The *P. falciparum* culture that contained mature gametocytes was used to generate ookinetes. Giemsa staining was used to verify the iRBC and ookinetes. Ookinetes were morphologically different from gametocytes under a microscope bright field (Fig. 3c). The mature ookinetes are crescent shaped with one sharp end and one blunt end, and have a distinct haemozoin pigment pattern. The cultured *P. falciparum* iRBC and ookinetes were fixed on glass slides and then incubated with FREP1 mixed with Chapel SA-3, followed by incubation with anti-FREP1 antibody, and fluorophore-conjugated secondary antibody. Since uninfected RBC do not have nuclei, DAPI was used to stain infected RBC. Results showed that the uninfected RBC could not bind FREP1 (Fig. 3d, 1^st^ row). Without the addition of Chapel SA-3, the FREP1 bound iRBC (asexual stage rings, sexual stage gametocytes) and mosquito midgut invasion form ookinetes (Fig. 3d, 2^nd^ row). Addition of the fungal extract abolished the interaction between FREP1 and iRBC as well as ookinetes (Fig. 3d, 3^rd^ row). Therefore, we concluded that Chapel SA-3 specifically inhibited the interaction between FREP1 and iRBC and ookinetes.

### Chapel SA-3 is nontoxic to human RBC, mosquito cell lines, and mosquitoes

**Figure 4:**
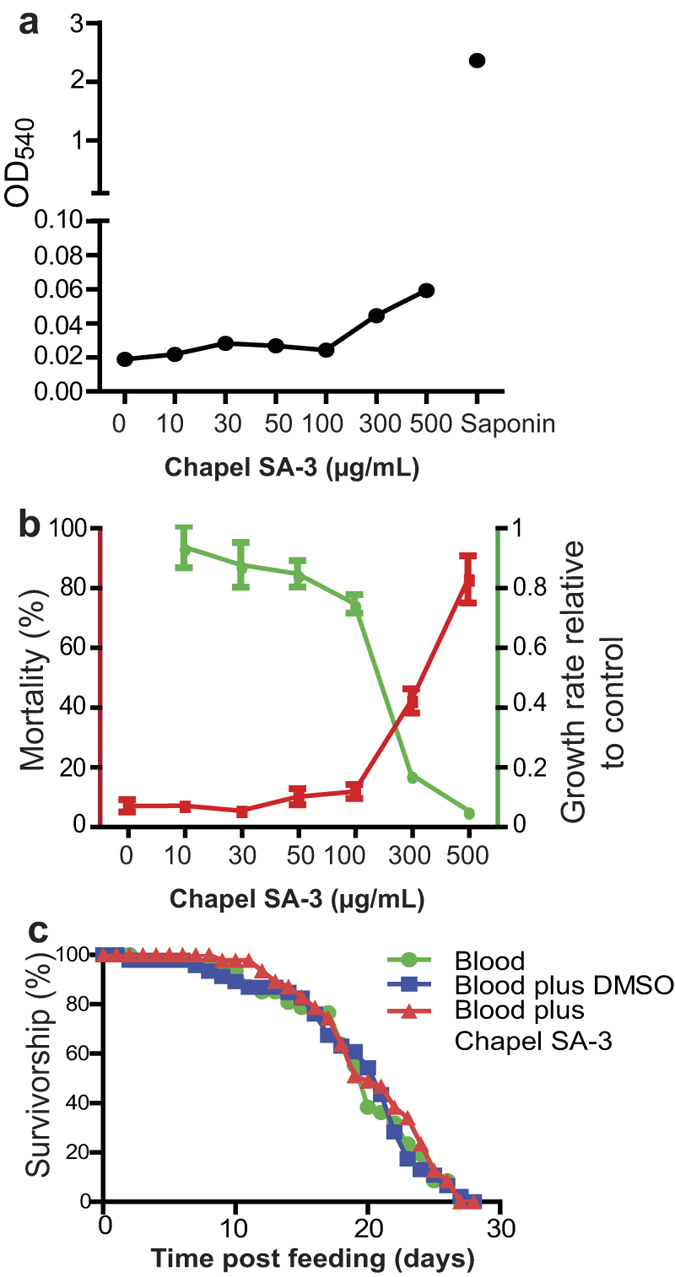
Chapel SA-3 extract is not toxic to cells and it does not affect mosquito lifespan. (**a**) The extract from isolate Chapel SA-3 did not lyse human blood at the concentration of 100 μg/mL or lower. (**b**) The extract from isolate Chapel SA-3 did not show apparent cytotoxicity to the mosquito cell line. Sua5B at the concentration of 100 μg/mL or lower. (**c**) There was no difference among the lifespan of adult mosquitoes that were fed with human blood, and human blood supplemented with 1% DMSO or 100 μg/mL Chapel SA-3 extract.

First, we analyzed the hemolysis activity of Chapel SA-3. Incubating different concentrations of Chapel SA-3 with 10% human RBC in PBS revealed that Chapel SA-3 did not cause detectable lysis of RBC at the concentration of 100 μg/mL or lower (Fig. 4a). Even at a high concentration of 500 μg/mL, Chapel SA-3 lysed much fewer RBC than the positive control (Saponin treated). Next, we tested the cytotoxicity of Chapel SA-3 on mosquito cells. A serial concentration of Chapel SA-3 was added into Sua5B cell culture medium. Two days later, alive and dead cells were counted. Results show no significant difference (*p* = 0.2) for Sua5B cells treated with Chapel SA-3 at a concentration of 100 μg/mL or lower (Fig. 4b), indicating that Chapel SA-3 is nontoxic to mosquito cells. Furthermore, we determined the effects of Chapel SA-3 on the lifespan of mosquitoes. Mosquitoes were fed with human blood or human blood supplemented with 1% DMSO or 100 μg/mL Chapel SA-3, and the numbers of surviving mosquitoes were counted every day. Our results showed that Chapel SA-3 did not kill mosquitoes immediately, and did not affect the mosquito lifespan (Fig. 4c). Collectively, Chapel SA-3 effectively prevented *P. falciparum* from infecting *An. gambiae* mosquitoes without introducing potential stress to mosquitoes, which will not exert any selective pressure on mosquito populations.

### Chapel SA-3 does not affect the development of gametocytes and ookinetes

Since mature *Plasmodium* gametocytes develop and fuse to form ookinetes that invade mosquitoes within 24 hrs after mosquitoes take an infected bloodmeal, we evaluated the effect of Chapel SA-3 on the differentiation of gametocytes into ookinetes. We diluted the iRBC containing mature stage V gametocytes with medium to stimulate the formation of ookinetes. A serial dilution of Chapel SA-3 in DMSO (0, 20, 100 μg/mL) was added into the culture. After 24 hrs of incubation, we quantitated the number of gametocytes and ookinetes under microscope. The conversion rate for the ookinetes in these *in vitro* culturing experiments is 0.12–0.16 (Table 1), consistent with previous publications (e.g. 0.12–0.42)^29^,^30^. The results showed no significant difference for gametocytes (*p* = 0.53) or ookinetes (*p* = 0.20) among the three treatments (Table 1), indicating that the candidate fungal extract does not interfere with the development of gametocytes into ookinetes. Collectively, our data supports the fact that Chapel SA-3 inhibits the parasite invasion in mosquitoes by disrupting the interaction between FREP1 and *P. falciparum*.

### Identification of the fungal species

**Figure 5:**
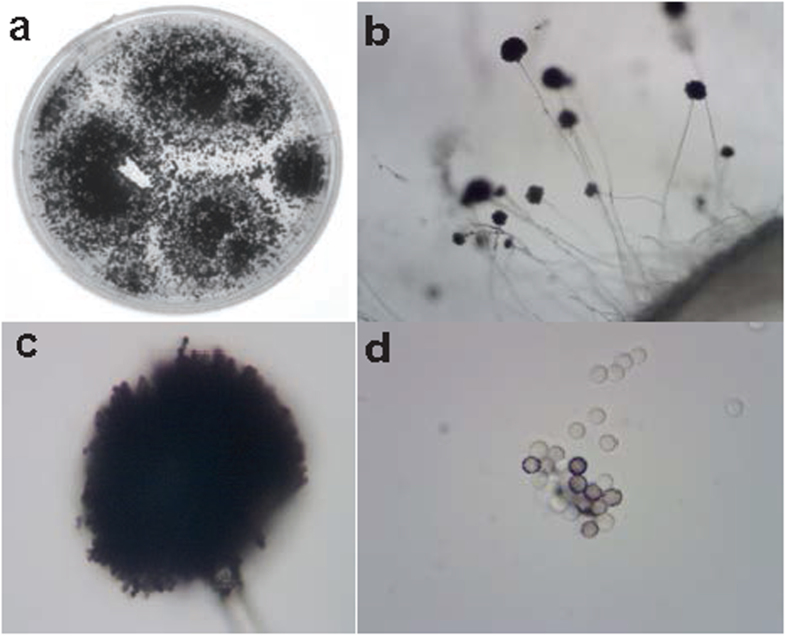
Isolate Chapel SA-3 colonies and spores. The fungus was grown on potato dextrose agar in a petri dish (**a**) and observed under the light microscope at 4X, and 40X magnification ((**b**,**c)**, respectively). The spores were separated and examined at 100X magnification (**d**).

We examined the morphology of the fungus using microscopy and identified the fungal species by molecular analyses. The fungus has very dark green, almost black spores. Based on the morphological observation shown in Fig. 5, we classified the fungus to the genus of *Aspergillus* Section *Nigri* (i.e., the black aspergilli). Thus, we sought to identify the fungus species by molecular analyses using PCR sequencing with standard internal transcribed spacer region (ITS) primers. We extracted the fungal genomic DNA, PCR-amplified the ITS DNA fragment using the fungal genomic DNA as templates, and sequenced the PCR product. The sequence was found to be 99% identical to *A. niger,* as well as its cryptic phylogenetic species *A. foetidus,* and *A. awamori*^31^ in the NCBI database. Considering the fungus morphology and its ITS PCR sequence, the fungus belongs to *A. niger* aggregate strains.

### Isolation of active compounds from the candidate fungus *A. niger*

**Figure 6:**
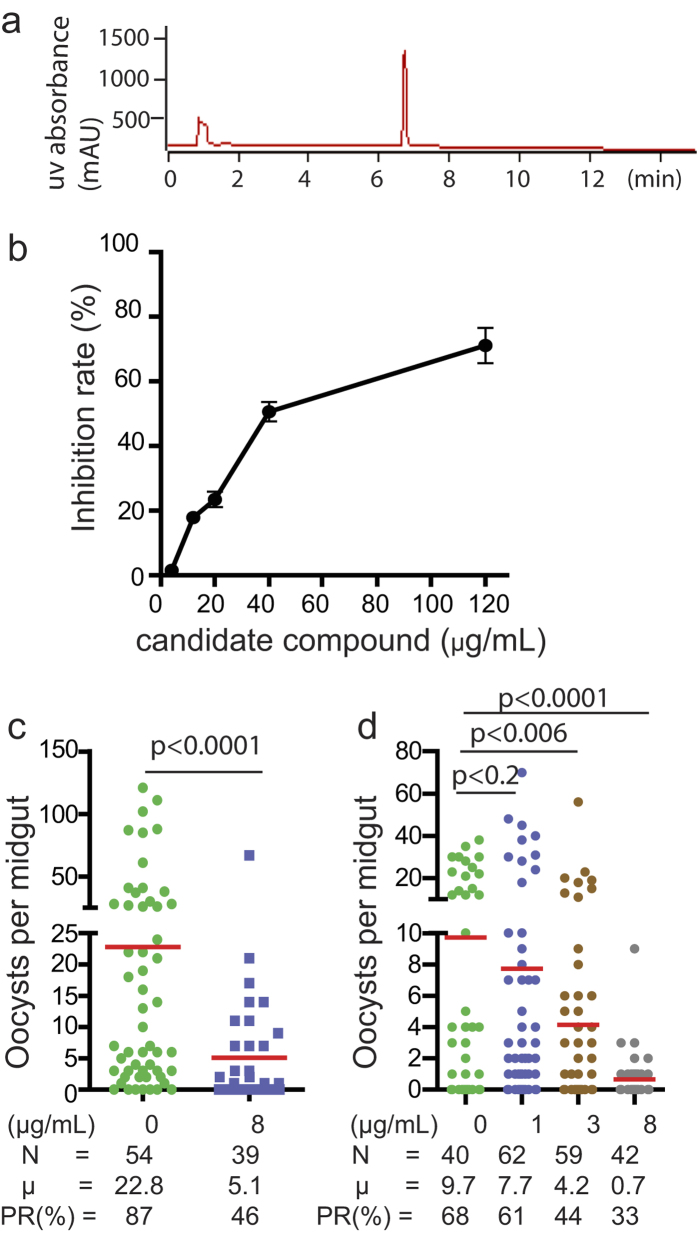
A pure compound was isolated from the Chapel SA-3 isolate’s extract that inhibited the FREP1-iRBC lysate interaction and *P. falciparum* infection in mosquitoes. **(a**) A bioactive pure compound showed a single peak in HPLC profile (PDA detection 190–400nm). (**b**) The candidate compound showed greater inhibition of FREP1 binding to iRBC lysate as the compound concentration increased. (**c**) The candidate pure compound (8 μg/mL) significantly inhibited *P. falciparum* infection. (**d**) The candidate pure compound’s inhibition of *P. falciparum* infection in mosquitoes displayed a dose-dependent pattern. N: the number of mosquitoes for each treatment; μ: the average number of oocysts per midgut; PR: infection prevalence in mosquitoes.

An extract consisting of 53.8 grams of crude natural-product-containing residue was prepared from an *A. niger* culture and fractioned by HP20ss flash column chromatography. The active fractions, which inhibited the interaction of FREP1-iRBC lysate were combined and subjected to HPLC as described in the Methods. One bioactive compound was purified. The purity of the fungal metabolite was confirmed by HPLC, wherein it eluted as a single peak at 6.8 min (PDA detection at 190–400 nm, Fig. 6a). The concentration-dependent inhibitory effects of the purified metabolite were analyzed over a range of 0 to 120 μg/mL for an ability to disrupt the interaction of FREP1-iRBC lysate using ELISA. Using the linear regression between logarithmic transformed dose and inhibition rates, we determined the IC_50_ of this compound to be 40 μg/mL (Fig. 6b). Next, we determined whether the pure compound was able to inhibit *P. falciparum* infection in mosquito midguts. Due to the much lower levels of endogenous FREP1 in mosquito midguts compared to the FREP1 in the ELISA assay, we tested the activity of the compound at a concentration well below the ELISA assay-derived IC_50_ value. For the experiment, 5 μL of pure compound (800 μg/mL) in DMSO was added into 495 μL blood containing 0.2% stage V *P. falciparum* gametocytes, and the mosquitoes were fed with SMFA. DMSO (5 μL aliquots) were used as vehicle-only controls. After dissecting the mosquitoes, significantly (p < 0.001) fewer oocysts were observed in the compound treated mosquito midguts than in the control samples (Fig. 6c). The results (Fig. 6d) also demonstrate that inhibition of *P. falciparum* infection occurred in a dose-dependent manner. As little as 3 μg/mL of the pure compound was capable of significantly reducing *P. falciparum* infection load in mosquitoes (p < 0.006), and reducing the number of oocyst by 56.7% and the infection prevalence rate by 35.3%.

### The active pure compound specifically prevents FREP1 from binding gametocytes and ookinetes

**Figure 7:**
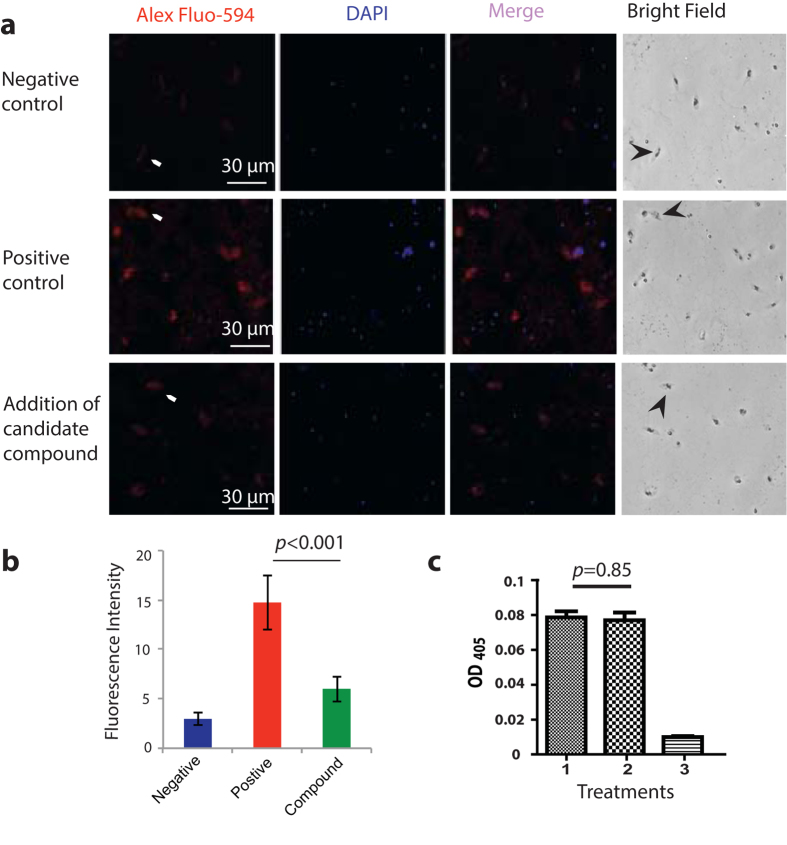
The pure candidate compound specifically prevents FREP1 from binding iRBC, gametocytes, and ookinetes. (**a**) The candidate compound specifically inhibited the binding of FREP1 protein to *P. falciparum* parasites as demonstrated by IFA. The first and second column detected FREP1 and parasite nuclei respectively. Merging column one and two generated the third column, which shows the co-localization of *P. falciparum* (nuclei) and FREP1 binding. The fourth column shows the bright views of the cells. No FREP1 signals were detected without adding FREP1 (1^st^ row, arrows points to gametocytes or ookinetes). Incubating FREP1 with *P. falciparum* gametocytes and ookinetes supplemented with 1% DMSO showed the co-localization of FREP1 and gametocytes or ookinetes (2^nd^ row). Addition of the candidate compound (40 μg/mL) reduced the interaction between FREP1 and gametocytes/ookinetes (3^rd^ row). The fourth row shows the bright field. Of note, many DAPI-positive dots that do not match with red spots are merazoites (free parasites), suggesting FREP1 does not bind merazoites well. (**b**) The intensity of red fluorescence indicated that the compound significantly prevented FREP1 from binding to gametocytes or ookinetes. (**c**) The candidate compound did not affect the ELISA reaction to detect FREP1, supporting the compound specifically interferes with FREP1-iRBC lysate interaction. Treatments: 1: FREP1 (7.5 μg/mL) plus DMSO (1%); 2: FREP1 (7.5 μg/mL) plus the candidate compound (40 μg/mL); 3: BSA (7.5 μg/mL) plus DMSO (1%).

We determined the specificity of the active compound in preventing FREP1 from binding to asexual stage parasites, gametocytes, or ookinetes using IFA. The *P. falciparum* gametocytes and ookinetes were fixed on glass coverslips using 4% paraformaldehyde in PBS. The FREP1 mixed with 40 μg/mL of the compound was incubated with the cells, followed by standard procedure of IFA. The DMSO (1%, v/v) without the compound was used as a positive control, and BSA replacing FREP1 as a negative control. Because asexual stage parasites are irrelevant to *Plasmodium* infection in mosquitoes, we focused on gametocytes and ookinetes. The fluorescence intensity values of gametocytes and ookinetes in three samples were: 3.97 ± 0.65 for negative control (Fig. 7a, 1^st^ row), 14.7 ± 2.73 for positive control (Fig. 7a, 2^nd^ row), and 6.01 ± 1.28 for the experimental group (Fig. 7a, 3^rd^ row). Of note, many DAPI-positive dots that do not match with red spots were merazoites (free parasites), suggesting FREP1 does not bind merazoites. Apparently, the compound significantly (p < 0.001) prevented FREP1 from binding to ookinetes (Fig. 7b). It is worth noting that the inhibition rate measured by IFA was consistent with the inhibition rate measured through ELISA with the same concentration of the compound (Fig. 6b).

Complementary to the above IFA approach, we also used the ELISA approach to confirm the specificity of candidate compound on the interaction between FREP1 and *P. falciparum* parasites, e.g. the compound does not inhibit unrelated molecule-molecule interaction. A plate was coated with FREP1. The anti-FREP1 antibody mixed with the candidate compound (40 μg/mL) was used to determine whether the compound interfered with the interaction between FREP1 and anti-FREP1 antibody or the interaction between anti-FRRP1 antibody and secondary antibody. Addition of the same amount of DMSO without the compound was used as a negative control. The result showed that the ELISA signal with the compound (Fig. 7c, column 2) was not significantly different (*p* = 0.85) from the negative control (Fig. 7c, column 1), suggesting that the candidate compound did not interfere with the interaction either between FREP1 and the anti-FREP1 rabbit antibody or between the 1^st^ antibody and 2^nd^ antibody. Collectively, these data supported the fact that the candidate compound specifically inhibits the interaction between FREP1 and parasites.

### Structural determination of the bioactive compound

**Figure 8:**
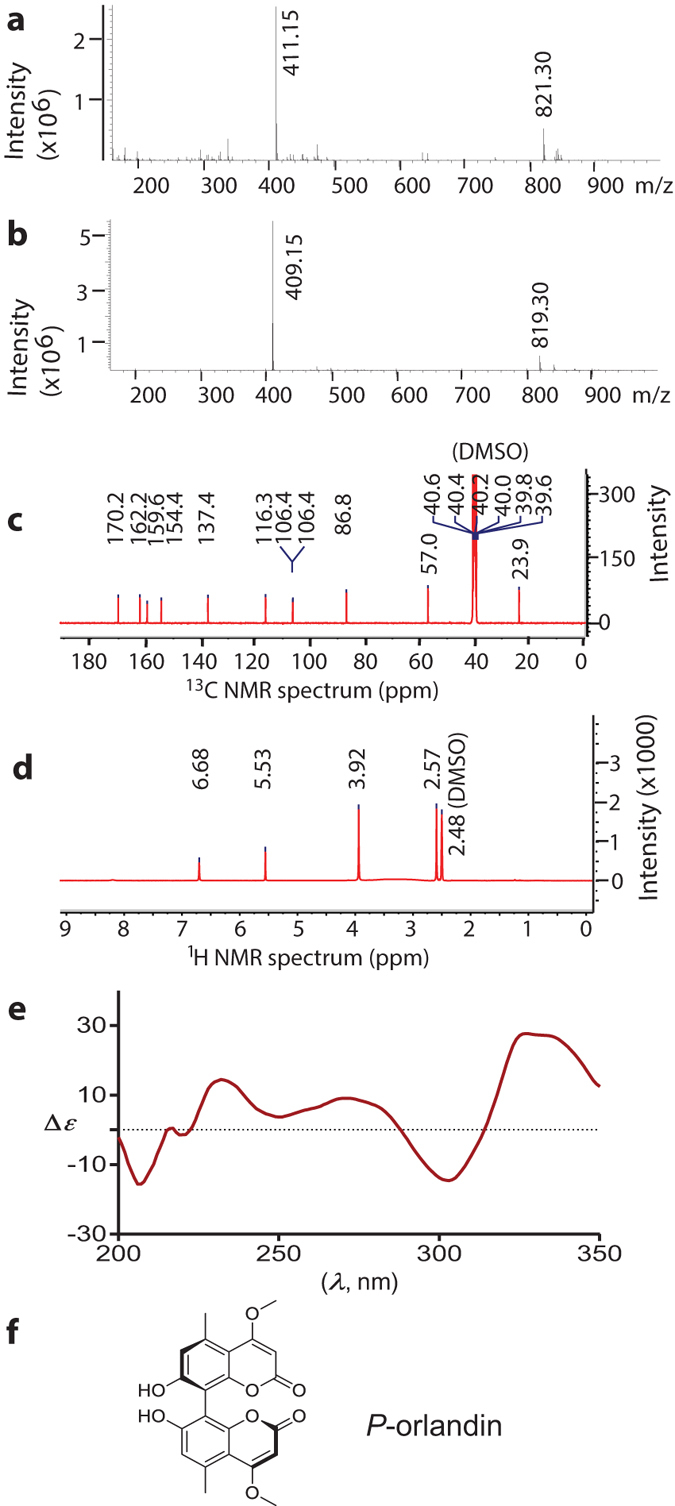
The candidate compound identified as *P*-orlandin. (**a**) Positive ion mode mass spectrum. (**b**) Negative ion mode mass spectrum. (**c**) ^13^C NMR spectrum. (**d**) ^1^H NMR spectrum. (**e**) Circular dichroism spectrum. (**f**) Chemical structure of *P*-orlandin.

Both positive and negative mode electrospray ionization mass spectrometry (ESIMS) were used to analyze the isolate. Based on ions observed at *m/z* 411.15 [M + H]^+^ and 821.30 [2M + H]^+^ (Fig. 8a), as well as 409.15 [M-H]^−^ and 819.30 [2M-H]^−^ (Fig. 8b), we rationalized that the compound possessed a molecular weight of 410 Da. Follow up analysis by high resolution ESIMS enabled us to unambiguously assign the compound a the molecular formula C_22_H_18_O_8_.

Since natural products produced by *Aspergillus* spp., including *A. niger*, have been extensively characterized, we proceeded with a dereplication strategy comparing the chemical data obtained for our purified metabolite with values published for other *Aspergillus*-derived natural products. An analysis of ^1^H and ^13^C NMR (100 and 400 MHz, respectively, DMSO-*d*_6_) data enabled us to identify a match to a known *Aspergillus* metabolite. The proton chemical shifts [*δ*_H_ 2.57, 3.93, 5.53, 6.68 (Fig. 8d)], as well as carbon chemical shifts [*δ*_C_ 23.9, 57.0, 86.8, 106.4, 106.4, 116.3, 137.4, 154.4, 159.6, 162.2, and 170.2 (Fig. 8c)] were identical to those published for the naturally-occurring bicoumarin, orlandin^32^. Based the NMR data and LC-ESIMS data, the metabolite was estimated to have >95% purity.

Although the planar structure of orlandin has been known for many years, the absolute configuration of the molecule arising from its axial chirality has not been reported. Fortunately, the absolute configurations of both naturally-derived and synthetically prepared samples of the structurally related metabolite kotanin^33^ have been examined in detail. This prior work enabled us to apply electronic circular dichroisms spectroscopy to compare data for orlandin and *P*-(+)-kotanin. A virtual match in the Cotton effects between data sets for the two compounds was obtained (Fig. 8e), indicating that orlandin is *P* configured (Fig. 8f).

### Orlandin is nontoxic to human blood cells and mosquito cell lines, and it does not affect the development of *P. falciparum* gametocytes and ookinetes

**Figure 9:**
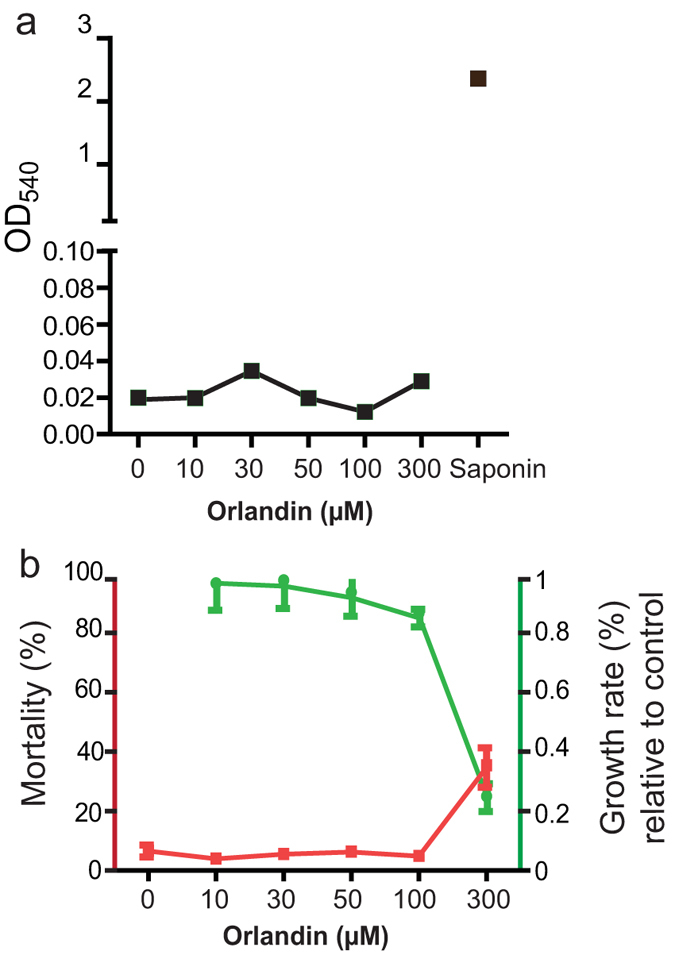
*P*-orlandin does not exhibit any cytotoxicity. (**a**) Orlandin did not lyse human blood cells at the concentration of 300 μM or lower. (**b**) Orlandin did not show apparent cytotoxicity to the mosquito cell line Sua5B at the concentration of 100 μM or lower.

Orlandin was previously reported to be nontoxic to mammals in chick and rat bioassays^32^. We further determined its general cytotoxicity. First we analyzed the hemolytic activity of orlandin. Different concentrations of orlandin were mixed with human RBC and incubated at 37 °C for 2 hrs. Saponin was used as a positive control to lyse blood cells. The OD_540_ of the supernatant was measured to quantify the hemolytic activity. As shown in Fig. 9a, orlandin did not lyse RBC at 300 μM or lower. Next, we examined whether orlandin is toxic to mosquito cell lines. A serial dilution of orlandin was incubated with *An. gambiae* cell line Sua5B for two days. The live and dead cells were recorded in each treatment. The wells without the addition of orlandin were used as a control. The results indicate that orlandin did not kill the cells (Fig. 9b, red line) or inhibit the cell growth (Fig. 9b, green line) at the concentration of 100 μM or below. Together, our data support the fact that orlandin is nontoxic to RBC and mosquito cells.

Finally, we examined the effect of orlandin on the development of *P. falciparum* gametocytes and differentiation of gametocytes into ookinetes. Twenty μM of orlandin was added into an ookinete culture containing *P. falciparum* stage V gametocytes. After 24-hour incubation, no significant difference in either gametocytes or ookinetes were found between orlandin and DMSO treatments (Table 2), indicating that orlandin neither is toxic to *P. falciparum* gametocytes nor affects the differentiation of gametocytes into ookinetes. In summary, we have purified and characterized the compound *P*-orlandin from *A. niger*, which inhibits *P. falciparum* infection in *An. gambiae* by interrupting FREP1-parasite interaction.

## Discussion

Due to the rapid spread of insecticide-resistant mosquitoes^7^ and drug-resistant *Plasmodium* parasites, the international campaign for malaria eradication requires novel approaches targeting novel molecules to control malaria. We previously reported that the mosquito protein FREP1 mediates *Plasmodium* infection in *An. gambiae*^16^ through its interaction with parasites in mosquito midguts^24^. Here we have developed a novel and efficient approach to block malaria transmission by targeting mosquito FREP1 protein using small fungal metabolite molecules.

First, we developed a novel high-throughput approach by targeting FREP1-iRBC lysate interaction to screen small molecules that potentially block malaria development in mosquitoes. This ELISA-based approach matches the criteria of a moderately high-throughput screening platform that is effective, low cost and reliable. As shown in the results, we obtained three positive hits out of 88 crude fungal extracts using the *in vitro* ELISA method. We further verified these activities using *in vivo P. falciparum* infection assays. All three candidates reduced the number of oocysts in mosquitoes and the nature of the *in vivo* activity matched the results of *in vitro* inhibition.

Parasite infection in mosquitoes is a major bottleneck in the *Plasmodium* life cycle. The number of asexual *P. falciparum* parasites in infected human blood is around 10^5^ to 10^6^ parasites per microliter of blood, and only ~450 asexual parasites per microliter differentiate to sexual gametocytes^34^. In mosquito midguts, and consistent with our *in vivo P. falciparum* infection, <5% gametocytes develop to ookinetes^34^ that are able to invade mosquito midguts. The smaller population size of gametocytes and ookinetes will delay the development of drug resistance in *Plasmodium*. Therefore, targeting parasite infection in mosquitoes is one of most effective approaches to prevent malaria transmission.

The ideal candidate compounds to control malaria are expected to be nontoxic to mammals or insects and inhibit parasite infection in mosquitoes by disrupting parasite invasion. Compounds exhibiting these features will be unlikely to exert selective pressure in mosquitoes and they are eco-friendly. We have shown that small molecules from *A. niger* can specifically interfere with FREP1-parasite interaction and prevents *P. falciparum* transmission to *An. gambiae*. *Aspergillus* is a member of the deuteromycetes fungi and has many medically important species. However, no metabolites from the known *Aspergillus* species have so far been reported to be active against malaria. Some of the *Aspergillus* species can release toxic mycotoxins^35^ including carcinogenic aflatoxins^36^; however, the strain used in these studies did not give evidence for the production of this or other mycotoxins^37^. Consistently, extracts from isolate Chapel SA-3 do not alter the lifespan of blood fed mosquitoes. According to a U.S. environmental Protection Agency report (http://www.epa.gov/biotech_rule/pubs/fra/fra006.htm), *A. niger* aggregates are not significant human pathogens.

Because the extract from *A. niger* was determined to be the best in the extract test pool at inhibiting FREP1-iRBC lysate interaction and *Plasmodium* infection in mosquitoes, we focused on demonstrating the principle of our proposed approach. Using bioassay-guided purification, the active compound was determined to be *P*-orlandin, and the collective results of the ELISA and IFA assays indicate that this fungal metabolite specifically interferes with the interaction between FREP1 and parasites. Consistent with previous reports that demonstrated orlandin is nontoxic to chicks and rats^32^, our data indicates that the compound is nontoxic to insect cells and mammal cells. Notably, orlandin showed good activity by inhibiting *P. falciparum* infection in mosquitoes. Even at doses as low as 3 μg/mL, orlandin significantly reduced the parasite infection intensity in mosquitoes by more than two fold.

There are two apparent methods to apply candidate compounds for malaria control: taken with traditional antimalarial drugs by patients or spraying bednets or inside the house. Complementary to traditional antimalarial drugs that focus on removing *Plasmodium* in human blood, taking this kind of anti-malaria transmission drug will maximize the elimination of malaria because the candidate small molecules in patient blood will block *Plasmodium* invasion in mosquitoes. On the other hand, spraying compounds to block malaria transmission will require large quantities of chemicals. Since it is relatively easy to culture fungi, it may be cost-effective to apply an orlandin-containing extracts inside of houses or bednets to confer resistance to malaria in mosquitoes, which is particularly important for the wide application in malaria endemic areas in developing countries.

In summary, we developed a novel approach by targeting the mosquito protein FREP1 to block malaria transmission and successfully identified the fungal metabolite orlandin as a candidate reagent to inhibit *P. falciparum* infection in *An. gambiae*. Compared to the current efforts focused on vaccine development^38^, transgenic^39^ or para-transgenic mosquitoes^40^,^41^, or insecticide development, targeting FREP1 with fungal metabolites for malaria control is novel, effective, and eco-friendly.

## Methods

### Ethical Statements

All animal experiments were performed strictly following the recommendations in the Guide for the Care and Use of Laboratory Animals of the US National Institute of Health. The Institutional Animal Care and Use Committee at the University of Oklahoma approved the procedure (R10-012).

### Rearing *An. gambiae* mosquitoes

*An. gambiae* G3 strain was reared in an insectary room maintained at 27 °C, 80% humidity with a 12-hr day/night cycle. Larvae were fed everyday with 0.1 mg ground fish food (KOI). Adult mosquitoes were maintained on 8% sucrose and fed with mouse blood for egg production.

### Fungus isolation and preparation of fungal extracts

The fungus responsible for producing orlandin was obtained from a soil sample collected near the parking area in the vicinity of St. Catherine Church on the island of Kauai, Hawaii. The soil sample was diluted in sterilized water and plated on multiple plate types for isolation and purification, as described previously^42^. The fungal isolate named “Chapel SA-3” was obtained from a 50% sucrose plate used to isolate osmophilic fungi that are capable of growth in an environment with very low water activity. Purified fungal isolates were cultured under solid-state conditions on a medium composed of Cheerios breakfast cereal supplemented with a 0.3% sucrose solution containing 0.005% chloramphenicol at room temperature (RT) for 4 weeks. The scale-up solid-phase cultures were pooled and extracted twice overnight with ethyl acetate, and the organic solvent was partitioned against water. The resultant organic layers were concentrated under vacuum. DMSO was added to dissolve fungal extracts.

### Culturing *P. falciparum* gametocytes and ookinetes

Asexual stage *P. falciparum* parasites (NF54 strain from MR4, Manassas, VA) were maintained in RPMI-1640 medium (Life Tech, Grand Island, NY) supplemented with 10% heat-inactivated (56 °C for 45 min) human AB^+^ serum (Interstate blood bank, Memphis, TN), 12.5 μg/mL hypoxanthine and 4% haematocrit (O^+^ human blood) in a candle jar at 37 °C^43^. To produce parasites at the gametocyte stage, the culture was diluted to 0.2–0.3% parasitemia with complete RPMI-1640 medium at 4% haematocrit. The medium was replaced every day to provide sufficient nutrients until day 15–17. Blood smears stained with Giemsa (Sigma-Aldrich, St. Louis, MO) were used to examine parasitemia or gametocytemia every other day under a light microscope. Following previous reports describing the generation of ookinetes^29^,^44^, the cultured *P. falciparum-*iRBC harboring stage V gametocytes were diluted 10-fold in RPMI-1640 supplemented with 20% human AB^+^ serum and 50 μg/mL hypoxanthine in a cell culture flask. The flask was then incubated at RT for 24 hrs to simulate the formation of zygotes and ookinetes. Stable and reproducible methods were established to obtain high yields of the sexual stage gametocytes and the mosquito invasion ookinetes using the *in vitro* culture system^29^,^45^,^46^.

### Expressing recombinant FREP1 protein in insect cells

To generate recombinant FREP1 similar to the endogenous FREP1, the complete coding sequence of *FREP1* was PCR-cloned into pIB/V5-His plasmid (Life Tech, Grand Island, NY) with the primer pair 5′-TCAAAGCTTCACCATGGTGAATTCATTCGTGTCG-3′ and 5′-ACTCTAGATTACGCGAACGTCGGCACAGTCGTG-3′, to generate the plasmid pIB-FREP1. After being amplified in *E. coli* DH5α, the plasmid was purified with an endotoxin-free plasmid preparation kit (Sigma-Aldrich). The cabbage looper ovarian cell-derived High Five cell line^47^ was used to express the recombinant FREP1 protein according to the user manual^48^. In brief, endotoxin-free recombinant pIB-FREP1 plasmid was mixed with Cellfectin® Reagent (1 μL Cellfectin/μg plasmid, Invitrogen, Grand Island, NY) in 5–6 mL Express Five® SFM medium (Invitrogen). The cells were cultured in 25 cm^2^ cell culture flasks (Greiner Bio-One, Monroe, NC) for 48 hrs at 27 °C. Medium and cells were separated by centrifugation at 300 × g for 5 min. The proteins in the medium were concentrated using Amicon® ULTRA-4 Centrifugal Filter Devices (Milipore, Billerica, MA) by centrifugation at 5,000 × g for 10 min and the protease inhibitors (Mini Tablets, EDTA-free, Thermo Scientific, Waltham, MA) were added to protect FREP1 from being degraded. Expression of FREP1 protein was determined by western blotting assays.

### Interaction assay between FREP1 and parasites using ELISA

The iRBC at 15–17-day culture and uninfected human RBC were collected and washed three times with RPMI-1640 at 300 × g for 4 min, and the cell pellets were then re-suspended in PBST (PBS containing 0.2% Tween-20). The cells were homogenized by ultra-sonication with 6 cycles of 10 sec pulse and 50 sec resting on ice for each cycle. The lysates were centrifuged at 8,000 × g for 2 min to remove insoluble materials and cellular debris. The protein concentration in the supernatant was measured using the Bradford protein assay^49^. An ELISA plate was coated with 100 μL of 2.0 mg/mL iRBC lysate per well overnight at 4 °C. Uninfected human RBC lysate that was at the same concentration of proteins was used as the control. After coating, the wells were blocked with 200 μL of PBS plus 0.2% bovine serum albumin (BSA) per well for 1.5 hrs at RT. After removal of the blocking solution, insect cell-expressed FREP1 (7.5 μg/mL) in blocking buffer (PBS plus 0.2% BSA) was added to each well and incubated for 1 hr at RT with gentle shaking. After washing three times with PBST, 100 μL rabbit anti-FREP1 polyclonal antibody (diluted 1:5,000 in blocking buffer, 1 μg/mL) was added to each well and incubated for 1 hr at RT. The purified anti-FREP1 antibody was obtained as described previously^24^. About 100 μL alkaline phosphatase-conjugated anti-rabbit IgG (diluted 1:20,000 in blocking buffer) was added to each well and incubated for 45 min at RT. The wells were washed three times with PBST between incubations. After washing, each well was developed with 100 μL pNPP substrate (Sigma-Aldrich) until the colors were visible, and absorbance at 405 nm was measured.

### Screening the fungal extracts that inhibit FREP1-iRBC lysate interaction by an ELISA-based assay

A 96-well ELISA plate was coated with 100 μL iRBC lysate (2 mg/mL protein). About 2 μg of fungal extract dissolved in 1 μL DMSO was mixed with 100 μL insect cell-expressed FREP1 (7.5 μg/mL) and added to each well. The iRBC lysate (100 μL) and the uninfected RBC lysate (100 μL) both supplemented with 1 μL DMSO were used as controls. The ELISA assays were performed as described above. The fungal extracts that reduced the ELISA signals were considered to contain active compounds that inhibit FREP1-iRBC lysate interaction. The inhibition rate was calculated with the equation, 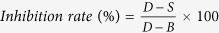, where *S* is the OD_405_ of a sample (iRBC lysate with addition of fungal extract), *B* is the OD_405_ of background control (uninfected RBC lysate, equivalent to complete inhibition), and *D* represents the OD_405_ of iRBC lysate with addition of DMSO (negative control, equivalent to no inhibition).

The ELISA approach was used in four replicates to confirm positive candidate fungal extracts or pure compounds that inhibited FREP1-iRBC lysate interaction and did not affect FREP1-antibody interaction or unrelated protein-protein interaction. The ELISA plate was coated at 4 °C overnight with 100 μL of insect cell-expressed recombinant FREP1 protein (7.5 μg/mL) or un-related protein with a 6xHistidine-tag. After blocking for 1 hr at RT, 1 μL identified fungal extract (10 mg/mL) or 1 μL pure compound (4 mg/mL) was mixed with 100 μL anti-FREP1 polyclonal antibody (diluted 1:5,000 in blocking buffer) or anti-His monoclonal antibody (Sigma-Aldrich, diluted 1:2,000 in blocking buffer), respectively. Finally, the corresponding secondary antibodies were used to probe primary antibodies and quantify the response through the standard ELISA assay. Antibody supplemented with 1 μL DMSO was used as a positive control and 100 μL BSA (7.5μg/mL in blocking buffer) supplemented with 1 μL DMSO was used as a negative/background control. The assay was repeated twice with four replicates per treatment.

### Indirect immunofluorescence assay to determine the inhibition specifictity of candidate fungal extracts and compounds on FREP1-parasite interaction

The 15–17-day cultured *P. falciparum* iRBC containing asexual stage parasites and gametocytes, and the cultured ookinetes were deposited onto premium cover glass slips to make blood smears. Before the smears were completely dehydrated, the semi-dry smears were fixed in 4% paraformaldehyde in PBS for 30 min at RT to keep the cell membrane intact. Then the cover glass was sequentially incubated in PBS that contained 10 mM glycine for 20 min and blocking buffer for 1.5 hrs at RT. After blocking, a candidate fungal extract was mixed with FREP1 (7.5 μg/mL protein, 100 μg/mL fungal extract or 40 μg/mL of a pure compound) in blocking buffer and incubated for 1 hr followed by sequential incubation with 4 drops of enhancer (Alexa Fluor ® 594 Goat Anti-rabbit SFX kit, Invitrogen) for 30 min, anti-FREP1 antibody (1:2,500 dilution in blocking buffer, 2 μg/mL) for 1 hr, and secondary antibody (Alexa Fluor ® 594 Goat Anti-rabbit SFX kit, Invitrogen; 1:1,000 dilution in blocking buffer) for 30 min. Between each incubation, the smears were washed three times with blocking buffer, 3 min each. At the end, the cover slip was rinsed in distilled water for 20 sec, and then coated with 4,6-diamidino-2-phenylindole (DAPI) (Sigma-Aldrich). Fifty μL of vectashield mounting media (Vector Laboratories, Burlingame, CA) was added onto the cover slip and mounted onto a slide. After incubation for at least 2 hrs in dark, the cells were examined under fluorescence microscopy (Nikon Eclipse Ti-S fluorescence microscope). Smears of uninfected human blood and the cultured *P. falciparum*-infected blood and ookinetes that were incubated with FREP1 supplemented with DMSO (1%) were used as controls. The fluorescence intensity was measured by Photoshop software (version CC 2014). To remove the noise or manipulation errors, the mean value of the background was subtracted from the mean fluorescence intensity of a target object.

### Analyzing the effect of a candidate fungal extract or a compound on *P. falciparum* infection in *An. gambiae*

The 15–17-day cultured *P. falciparum* iRBC containing 2–3% gametocytes at stage V were collected and diluted with fresh O^+^ type human blood that was mixed with the same volume of heat-inactivated AB^+^ human serum. The final concentration of the stage V gametocytes in the blood was around 0.2%. Then, three dilutions of a candidate fungal extract were individually added to the blood, and the final concentrations of the fungal extract were 0, 20, and 100 μg/mL. SMFA^16^,^50^ was performed to feed ~100 3-day old female mosquitoes for 15 min, and the engorged mosquitoes were maintained with 8% sugar in a BSL-2 insectary (28 °C, 12-hr light/dark cycle, 80% humidity). The midguts were dissected 7 days post-infection and stained with 0.1% mercury dibromofluresein disodium salt in PBS. The oocysts were counted using light microscopy, and non-parametric statistical analyses (Mann-Whitney-Wilcoxon test) were used to determine the difference between the experimental and control groups. To test the effect of a pure compound, the fungal extracts were substituted with the pure compound, and the final concentrations of the compound were 0, 1, 3, and 8 μg/mL. All other procedures were the same.

### Analyzing the effect of a candidate fungal extract or a compound on mosquito fitness and ookinete development

To determine whether the fungal extract affects adult mosquito fitness, fifty 2-day-old female mosquitoes were fed with human blood and the blood supplemented 1% DMSO or 100 μg/mL of the fungal extract respectively. The number of the dead mosquitoes was recorded and removed daily until all of the mosquitoes died.

To determine the effect of a fungal extract or a pure compound on the survival and the development of *P. falciparum* gametocytes and ookinetes, we cultured 17-day *P. falciparum* parasites containing ~3% stage V gametocytes in culture medium mixed with a serial dilution of the fungal extract (0, 20 and 100 μg/mL) or the compound (0 and 8 μg/mL) and quantitated the number of gametocytes and ookinetes 24 hrs post-incubation by blood smears stained with Giemsa. A total of 30 microscopic views were randomly picked up for analysis and triplicates for each treatment were performed. The assays were repeated twice and the one-way analysis of variance (ANOVA) and t-test was used to analyze the data respectively.

### Analysis of the general cytotoxicity of a fungal extract and a candidate compound

To determine whether the candidate fungal extract can induce hemolysis, 1 μL of a fungal extract in DMSO (with concentrations of 0, 1, 3, 5, 10, 30, 50 mg/mL) or a pure compound (0, 1, 3, 5, 10, 30 mM) were mixed with 99 μL blood (10% haematocrit in PBS). After incubation at 37 °C for 120 min, 50 μL of the supernatant was transferred to a new 1.5 mL tube. After centrifugation for 4 min at 500 × g, the supernatant was transferred into a 96 well plate and OD_540_ was read. To remove the background absorbance of a candidate fungal extract or a candidate compound, the final absorbance value was obtained by subtracting the OD_540_ of the corresponding concentration of the fungal extract or compound diluted with H_2_O from the OD_540_ of treated wells. Saponin, at a final concentration of 0.8 mg/mL that can lyse RBC, was used as a positive control. Each treatment included four replicates and the assay was repeated twice.

To examine the cellular toxicity of a fungal extract or a compound, a mosquito cell line (Sua5B) was diluted to 5 × 10^5^ cells/mL in S2 medium (Schneider’s *Drosophila* medium, 5% heat inactivated fetal calf serum, and 1% penicillin-streptomycin antibiotic) and mixed with a fungal extract or a compound (1:100, v/v) to obtain the final concentrations of 0, 10, 30, 50, 100, 300, 500 μg/mL for fungal extract or 0, 10, 30, 50, 100, 300 μM for a pure compound. The cells were then incubated at 27 °C for 2 days in a 96 well plate. After incubation, the cells were stained with trypan blue and counted with hemocytometer. Mortality and the growth rate of the cells relative to the control (no fungal extracts or compound) were calculated for each treatment.

### Identification of the fungal species by morphology and molecular approaches

The fungus was cultured in a Petri dish containing potato dextrose agar at RT for three days or until the spores were visible, and the fungus was then morphologically analyzed by light microscopy. To examine the fungal spores, we deposited the compressed spores onto a glass slide for observation. The fungus was further identified through PCR using ITS primers^51^. To perform DNA sequencing, the fungus was grown in a shake culture in a glass tube containing a 0.3% sucrose solution to avoid the formation of the copious amount of black spores. A small amount of the mycelium was taken and rinsed by centrifugation at 15,000 × g in 400 μL sterilized water. The mycelium was then suspended in 100 μL of sterilized water and 1 μL was taken for PCR. The DNA fragment was amplified with the ITS1 (TCCGTAGGTGAACCTGCGG) and ITS4 (TCCTCCGCTTATTGATATGC) primers, and purified with Qiagen DNA purification kit (Valencia, CA). Sanger approach was used to sequence the PCR product.

### Isolation and identification of active compounds from a candidate fungal extract

The fermentation products were homogenized and extracted with ethyl acetate (1:1 v/v) twice. The extracts were combined, dried under vacuum, and dissolved in DMSO. The extract was applied to a HP20ss flash column, and eluted with a gradient of MeOH-H_2_O (20:80, 40:60, 60:40, 80:20, 100:0) and MeOH-DCM (50:50) to yield six fractions. The ability of each fraction to inhibit FREP1-iRBC lysate interaction was determined using ELISA assays. The bioactive fractions 3 and 4 were combined and the compounds further separated by preparative HPLC (Waters, 1525 binary HPLC pump coupling to a 2998 PDAD) on Gemini C_18_ column (250 mm × 20.2 mm, 5 μm, Phenomenex) using a gradient of MeOH-H_2_O with 0.1%HCOOH (20:80–100:0), followed by semi-preparative HPLC (Gemini C_18_ 250 mm × 10 mm, 5 μm, Phenomenex) using an isocratic MeCN-H_2_O with 0.1%HCOOH (32:68) to obtain the pure bioactive compound. LC-MS (Shimadzu UFLC system coupling to a Shimadzu single quadrupole mass spectrometer) with Kinetex C_18_ column (3.0 mm × 75 mm, 2.6 μm, Phenomenex) using a gradient of MeCN-H_2_O with 0.1% HCOOH (10:90–100:0), and 1D nuclear magnetic resonance (NMR, Varian Unity Inova 600 MHz) spectra were used to determine the molecular mass and planar structure of the active compound. Finally, we measured its circular dichroism (CD) spectrum (AVIV circular dichroism spectrometer model 202-01) and compared with the published CD spectrum of its analogue to determine the absolute configuration of the bioactive compound.

## Additional Information

**How to cite this article**: Niu, G. *et al.* Targeting mosquito FREP1 with a fungal metabolite blocks malaria transmission. *Sci. Rep.* **5**, 14694; doi: 10.1038/srep14694 (2015).

## Figures and Tables

**Table 1:** Chapel SA-3 does not affect the development of gametocytes or ookinetes.

Concentration (μg/mL)	Gametocyte (%)	*P*-value(ANOVA)	Ookinete (%)	*P*-value(ANOVA)
0	2.60 ± 0.30		0.33 ± 0.04	
20	3.01 ± 0.20	*0.53*	0.48 ± 0.06	*0.20*
100	2.67 ± 0.27		0.43 ± 0.05	

**Table 2:** Orlandin does not affect the development of gametocytes or ookinetes.

Treatments	Gametocyte (%)	*P*-value (*t*-test)	Ookinete (%)	*P*-value (*t*-test)
DMSO	1.23 ± 0.30	0.73	0.10 ± 0.06	0.72
Orlandin	1.34 ± 0.13		0.15 ± 0.05	
